# Lessons Learned from a Small Pediatric Continuous Renal Replacement Therapy Program

**DOI:** 10.1155/2021/6481559

**Published:** 2021-11-17

**Authors:** Tanya Holt, Olivia Griffin, Amelie Cyr, Rebecca Brockman, Laura Wihak, Gregory Hansen

**Affiliations:** ^1^Jim Pattison Children's Hospital, Pediatric Intensive Care, 103 Hospital Drive, S7N 0W8, Saskatoon, Saskatchewan, Canada; ^2^College of Medicine, University of Saskatchewan, 107 Wiggins Rd, S7N 5E5, Saskatoon, Saskatchewan, Canada

## Abstract

Continuous renal replacement therapy (CRRT) has become a pillar of care in pediatric intensive care units (PICUs) over the past few decades. Quality indicators (QIs) have been evaluated that reflect safe and accountable CRRT. However, there is a paucity of data on outcomes and QIs in smaller-volume CRRT programming. The purpose of this retrospective study was to evaluate the efficiencies, effectiveness, and outcomes of a small-volume CRRT program. Eighty-two patients received CRRT over a 13-year period, and 79% survived to discharge. Sepsis or nonseptic shock (*n* = 11 (22%) versus *n* = 6 (50%); *p* value = 0.004) and time to CRRT initiation after PICU admission (1.1 versus 5.0 days; *p* value = 0.005) were independent predictors for mortality. The program also had positive outcomes for QIs related to CRRT efficiency and time of initiation, dosing delivery, and rate of adverse events. This study is important as it illustrates the opportunity that smaller centers have to initiate CRRT programming and provide safe and effective care.

## 1. Introduction

Continuous renal replacement therapy (CRRT) has become the mainstay of renal replacement therapy in pediatric intensive care units (PICUs) over the past three decades [1, 2]. Advancements in technology, equipment, and patient care-related guidelines have resulted in CRRT being the preferred technique to manage critically ill children with acute kidney injury (AKI) and fluid overload [2, 3]. CRRT has adapted into a readily accessible bedside therapy with unique advantages for hemodynamically unstable patients as compared to intermittent hemodialysis and peritoneal dialysis [1, 3].

Despite the evolving sophistication of this therapy in the PICU setting, there remains wide practice variation in its application. This is in part related to a dearth of evidence concerning the time of CRRT initiation, dose prescription, optimal mode, and time of discontinuation. A large prospective pediatric CRRT registry reported no clear associations between CRRT dose and mortality [2]. A later study found that percentage of volume overload at CRRT initiation was a single indicator associated with worse outcomes [4]. A recent modified Delphi process identified and prioritized quality indicators (QIs) that could be used as a safety and effectiveness benchmark for CRRT programs [5,6]. Example indicators included filter life, downtime, time to initiation, dose delivered, and fluid management. A large single-center retrospective evaluation of a pediatric CRRT program used similar metrics as related to PICU 28-day and 60-day survival and survival to discharge [7].

Saskatchewan's PICU admits just under 500 patients per year, and approximately 1.5% require CRRT. Thirteen years ago, a CRRT program was developed in response to population demographics, unmet needs in patients with complex medical backgrounds and/or primary AKI, and the evolving literature related to sepsis, volume overload, and advantages of CRRT in the critical care population. More specifically, 10–15% volume overload was used as a trigger to consider initiation of CRRT, as increasing volume overload was shown to have a direct association with mortality [2]. Prior to this, critically ill children requiring dialysis had to leave the province for extended periods. Medical expertise and leadership within the pediatric intensive care unit (PICU) was identified, synergies with the adult CRRT program were created, and bedside practitioners were trained through Gambro's^®^ (Baxter Healthcare Corporation, Chicago, IL) orientation program and bedside mentorship with adult ICU CRRT practitioners. The initial patient volume projection was 3-4 patients per year, but this proved to be an underestimation and resulted in the need for procurement of a dedicated pediatric machine.

CRRT is often reserved for the most unstable patients in the PICU and should be delivered in a consistent, safe, and high-quality manner regardless of the program size. Identification of QIs that reflect safe and accountable pediatric CRRT in a small program is crucial, yet unexplored. The purpose of this retrospective study was to evaluate the efficiencies, effectiveness, and outcomes of a small-volume CRRT program.

## 2. Materials and Methods

This retrospective case comparison within a cohort study examined data from pediatric patients requiring CRRT between October 2007 and December 2020. The University of Saskatchewan Research Ethics Board granted ethics approval for the study. The inclusion criteria included all patients <17 years admitted to PICU who required urgent CRRT during their stay. Exclusion criteria included stable patients with a known preexisting condition requiring intermittent CRRT.

### 2.1. Program

The Saskatchewan CRRT program uses the PRISMAFlex^®^ (Baxter Healthcare Corporation, Chicago, IL) machine. The choice of filters vary between the HF20, ST60, and ST150 for <11 kg, <30 kg, and >30 kg, respectively. The primary modes of CRRT used are Continuous Veno-Venous Hemodiafiltration (CVVHDF) and Continuous Veno-Venous Hemodialysis (CVVHD). The dialysate and replacement fluid options are Prism0CAL and Prism0SOL used with and without citrate anticoagulation, respectively.

### 2.2. Variables

Demographic variables included age, biologic sex, weight, height, and body surface areas. Clinical variables included final diagnoses, hospital and PICU admission creatinine, urea, potassium, urine output levels, percentage of volume overload, pediatric injury severity scoring (PELOD-2, PRISM), categorizing AKI (KDIGO and pRIFLE criteria), and requirements for inotropes and conventional mechanical ventilation. CRRT variables included catheter placement, time of initiation (time from PICU admission to CRRT initiation days), percentage of dose delivered (actual CRRT dose delivered/24-hour prescribed dose in ml/kg/hr), filter life (24-hour time on CRRT in %), duration (total duration on CRRT in days), presence of citrate lock, and complications including bleeding, central line-associated bloodstream infections, and thrombocytopenia. Citrate lock was defined as citrate accumulation as reflected by a rising total to ionized calcium ratio (Ca/Ca++) [8]. Outcomes included end-stage kidney disease (ESKD) defined as need for chronic dialysis and/or transplantation; length of hospital/PICU stay; and mortality.

### 2.3. Statistics

The primary purpose of this study was to evaluate predictors for mortality in PICU patients on CRRT. Descriptive statistics were used to compare survivors with nonsurvivors. Univariate analyses included a two-tailed Fisher's exact test for comparing proportions of categorical data. Difference of means with 95% confidence intervals was calculated for continuous data summarized by means and standard deviations.

Sepsis or nonseptic shock, PELOD-2 score, 12-hour postadmission PRISM score, admission percent volume overload, and time to CRRT initiation were considered for multivariate analysis. Independent variables were considered for binomial logistic regression if *p* value was <0.1. Significance level was considered at 0.05.

## 3. Results

A total for 82 patients received RRT during a 13-year period. Nineteen were excluded from analysis as ten received intermittent renal replacement therapy and another nine received CRRT but had incomplete charting. Of the remaining 63 patients, 51 survived (81%) to hospital discharge, and their demographics are summarized in Table 1. The survival rate of those patients with incomplete charting was 77%.

Indications for CRRT, CRRT metrics, and admission clinical or laboratory data are summarized in Table 2. The diagnosis of either sepsis or nonseptic shock (*n* = 11 (22%) vs. *n* = 6 (50%); *p* value = 0.004) or time to CRRT initiation after PICU admission (1.1 vs. 5.0 days; *p* value = 0.005) were independent predictors for mortality. Hemocatheters were preferentially inserted at the bedside by intensivists (*n* = 44; 70%) and into an internal jugular vein (*n* = 35; 56%). No circuits were blood primed, including for neonates. The PRISMAFlex^®^ HF20 filter (Baxter Healthcare Corporation, Chicago, IL) requires only 60 ml of extracorporeal blood volume in a set and allows for greater hemodynamic stability at CRRT initiation and often precludes the need for blood primes in the neonatal cohort. Regional anticoagulation began with 4% citrate (*n* = 10; 16%) but was then exclusively transitioned to ACDA (*n* = 51; 84%). Location of the hemocatheter was associated with a longer filter life (internal jugular 0.87 vs. femoral 1.20 filters/day; *p* value = 0.06).

There were differences between survivors and nonsurvivors in regard to PICU management. Nonsurvivors more likely required conventional mechanical ventilation (*n* = 12 (100%) vs. n = 36 (69%); *p* = 0.03) and inotropes (*n* = 12 (100%) vs. *n* = 32 (62%); *p* = 0.01) and had higher 12-hour postadmission PRISM (22.3 vs. 17.7; *p* = 0.03) and PELOD-2 (9.6 vs. 7.1; *p* = 0.08) scores.

Outcomes are summarized in Table 3. A diagnosis of sepsis or nonseptic shock (odds ratio 8.6; 95% CI 1.45, 50.78; *p* = 0.02) and time to CRRT initiation (odds ratio 1.3; 95% CI 1.05, 1.61; *p* = 0.02) were significant predictors for mortality.

## 4. Discussion

In this retrospective case comparison within a cohort study of a small CRRT program, our major finding was a hospital mortality rate of 19%. Our mortality rate compares well with others published (29.8–42%) [9,10], but it should be noted that none were receiving concurrent extracorporeal life support. However, neonates who were usually over-represented with higher mortality rates [10] were lower in our cohort at 17%. Together, these outcome data are encouraging for a small and burgeoning CRRT program, even at the extremes of age. Sepsis, nonseptic shock, and prolonged time to CRRT initiation were multivariate predictors for mortality, which mirror those reported in a recent study [9,10]. Although septic shock has high mortality rates at baseline, its concomitant higher incidence of fluid overload [11,12] likely represents both a modifiable clinical factor and marker of disease severity.

One of the most challenging aspects of providing optimal CRRT is the decision to initiate [2]. Modem et al. [13] evaluated the time between PICU admission and CRRT initiation and reported that two versus 3.4 days were associated with survivors and nonsurvivors, respectively, independent of the total duration of CRRT therapy. Our data were consistent with these observations as two contributing factors emerged that reflected the encouraging efficiencies related to our program and patient CRRT initiation. First, early recognition of AKI and/or percentage of fluid volume overload as a trigger for initiation has been shown to improve overall outcomes [2, 4]. Our mean percent volume overload at CRRT initiation was 8.5%, which was lower than the 10% threshold previously identified that increased mortality [4]. Second, vascular access is a critical step to timing, initiation, and ongoing dose delivery of CRRT. Seventy percent of our program's hemovascular catheters were percutaneously placed with ultrasound guidance at the bedside. This prevents initiation of CRRT being dependent on the coordination, communication, and patient transfers related to interventional radiology or general surgical insertions.

The goal of CRRT is effective and timely solute, fluid, and acid-base control [14]. Quality indicators including filter lifespan, 80% effluent dose and ultrafiltrate rate of what has been prescribed, and less than 20% interruptions of CRRT for every delivery day have been suggested for effective CRRT [5, 6]. Our average filter lifespan was over 24 hr, with interruptions generally related to off-unit procedures. The results for CRRT dosing, fluid removal, and downtime over 24 hr were also compliant with the quality indicators, suggesting an overall effectiveness of CRRT delivery.

Absence of catheter line-associated blood stream infections (CLABSI) and bleeding are high-validity QIs for CRRT delivery [5]. Although our rate of thrombocytopenia was 27%, there were no bleeding episodes in our cohort. Moreover, we had no incidences of CLABSI. This positive result may be related to our standardized policy on hemocatheter utilization and access by specialized nurses only. Hemocatheter use was dedicated to CRRT delivery and rarely for concomitant therapeutic plasma exchange. If there was circuit downtime, the hemocatheter was citrate locked and when reaccessed, was done under sterile precautions.

For smaller PICUs, it may be challenging to initiate and deliver a high-quality and consistent CRRT program. Several factors have contributed to our program's quality and success. We started the program with a small, dedicated group of experienced PICU nurses in order to ascertain a critical volume of experience each year. Standardized order sets and guidelines for CRRT dosing and line placement allowed consistent practice within our physician group. A collaborative approach was also taken with our nephrology colleagues which supported successful transitions and weaning off CRRT for follow-up or ongoing chronic RRT. Finally, early recognition of potential CRRT patients related to percent volume overload and AKI criteria may have been an important contributor to our timely initiations and positive outcomes. Our PICU was transitioned to have percent fluid volume overload reported on all patients in daily rounds to recognize the increased risk.

The major limitation of our study was the smaller sample size. Moreover, there was a lack of chart accessibility for nine patients. A contributing factor was that during the data collection period, our hospital transitioned from paper to electronic charting. Regardless, the survival rate for these patients was 78% (*n* = 7), mirroring what was reported for our larger cohort. Secondly, as the study retrospectively was conducted over a thirteen-year period, CRRT practice, bedside practitioners, and intensivists changed or evolved. Finally, while older charts may have missing or unavailable information leading to bias, our standardized CRRT documentation has been relatively unchanged.

## 5. Conclusion

The frequency of AKI accompanied by concomitant volume and hemodynamic vulnerabilities make CRRT a crucial therapeutic program for any PICU. QIs have been established that reflect safe and accountable CRRT delivery. This study is important as it illustrates that smaller PICUs can initiate safe and effective CRRT programming that leads to positive outcomes.

## Figures and Tables

**Table 1:** Patient demographics.

	Survivors (*n* = 51)	Nonsurvivors (*n* = 12)
Age, years^a^	7.00 (13.6)	6.1 (11.6)
Height, cm^a^	138.5 (83.5)	112.8 (97.8)
Weight, kg^a^	26.1 (53.1)	23.0 (57.6)
Body surface area, m^2a^	1.0 (1.2)	0.8 (1.3)

^a^Median (interquartile range).

**Table 2:** Renal and continuous renal replacement metrics.

	Survivors (*n* = 51)	Nonsurvivors (*n* = 12)	*p* value
Indications for CRRT^a^			0.07
Renal failure	19 (37)	1 (8)	
Sepsis	6 (12)	4 (33)	
Nonseptic shock	5 (10)	4 (33)	
Neonatal	5 (10)	1 (8)	
Oncologic	6 (12)	2 (17)	
Overdose	8 (16)	0	
Other	2 (4)	0	

Sepsis or nonsepsis shock diagnosis^a^	11 (22)	8 (50)	0.004
Admission KDIGO score^a^			0.52
0	8 (16)	0	
1	5 (10)	1 (8)	
2	18 (35)	5 (42)	
3	20 (39)	6 (50)	

Admission pRIFLE score^a^			0.45
Normal	8 (16)	1 (8)	
Risk	8 (16)	2 (17)	
Injury	8 (16)	4 (33)	
Failure	25 (49)	4 (33)	
Loss	0 (0)	0 (0)	
End stage	1 (2)	1 (8)	

Creatinine, *μ*mol/L^b^			
Hospital admission	397 (570)	295 (468.8)	0.58
PICU admission	394 (508)	300 (410)	0.55

Urea, mmol/L^b^			
Hospital admission	18.1 (16.8)	14.6 (14.2)	0.51
PICU admission	20.8 (15.7)	19.0 (14.4)	0.73

Urine output, ml/kg/hr^b^			
Hospital admission	0.79 (0.94)	0.58 (0.82)	0.47
PICU admission	1.34 (1.99)	1.22 (1.93)	0.76

Potassium, mmol/L^b^			
Hospital admission	4.8 (1.2)	4.8 (1.3)	0.94
PICU admission	4.7 (1.1)	4.6 (1.2)	0.85
Percent volume overload^b^	7.4 (7.2)	12.0 (8.6)	0.06
Actual CRRT dose delivered/24-hour prescribed dose (ml/kg/hr)^b^	0.85 (0.2)	0.95 (0.1)	0.15
UF removed 24 hr/UF prescribed 24 hr, %^b^	1.3 (0.86)	1.5 (0.75)	0.47
24 hr time on CRRT, %^b^	0.91 (0.1)	0.95 (0.04)	0.10
Citrate lock^a^	18 (35)	3 (25)	0.74
Duration of CRRT, days^b^	5.9 (5.1)	7.1 (6.7)	0.51
Time from PICU admission to CRRT initiation, days^b^	1.1 (2.6)	5.0 (5.3)	0.0005

^a^Number (proportion); ^b^mean (standard deviation). KDIGO: kidney disease improving global outcomes; pRIFLE: pediatric risk, injury, failure, loss, end stable renal disease; UF: ultrafiltrate.

**Table 3:** Patient outcomes.

	Survivors (*n* = 51)	Nonsurvivors (*n* = 12)	*p* value
Complications^a^			
CLABSI	0	0	
Bleeding	0	0	
Thrombocytopenia	17 (33)	6 (50)	0.28
Catheter dysfunction	1 (2)	1 (8)	0.26
Transplant^a^	2 (4)	0	—
Hospital length of stay, days^b^	39.4 (38.9)	19.8 (20.9)	0.1
PICU length of stay, days^b^	17.7 (19.1)	14.7 (16.2)	0.6

^a^Number (proportion); ^b^mean (standard deviation).

## Data Availability

Data are available upon request to the corresponding author.
